# Maternal trait personality and breastfeeding duration: the importance of confidence and social support

**DOI:** 10.1111/jan.12219

**Published:** 2013-08-06

**Authors:** Amy Brown

**Affiliations:** ^1^ Department of Public Health and Policy StudiesCollege of Human and Health SciencesSwansea UniversityUK

**Keywords:** breastfeeding, confidence, midwives, nursing, paediatrics, self‐efficacy, social support, trait personality

## Abstract

**Aim:**

To explore associations among breastfeeding duration, maternal personality and maternal attitudes and experiences of breastfeeding.

**Background:**

Understanding influences on breastfeeding initiation and duration is critical to increasing breastfeeding rates and supporting new mothers. Maternal characteristics such as self‐efficacy, knowledge and confidence are known to enable women to breastfeed, but little is known about the influence of maternal trait personality on breastfeeding.

**Design:**

An exploratory cross‐sectional survey.

**Method:**

A total of 602 mothers with an infant aged 6–12 months old completed a self‐report questionnaire examining maternal trait personality, breastfeeding duration and attitudes and experiences of breastfeeding. Data were collected between March–June 2009.

**Results:**

Mothers who reported high levels of extraversion, emotional stability and conscientiousness were significantly more likely to initiate and continue breastfeeding for a longer duration. Attitudes and experiences significantly associated with these personality traits such as perceived difficulties and lack of support may explain these patterns. For example, characteristics associated with introversion and anxiety may prevent women from seeking support or challenging negative attitudes of others at this critical time.

**Conclusion:**

Understanding the influence of maternal personality may thus be a useful tool in antenatal support to recognize women who may need extra, directed support while facilitating discussion of potential barriers to breastfeeding.

**Why is this research or review needed?**

- Interventions are needed to increase levels of breastfeeding in the UK and other countries.
- Decision to initiate and continue breastfeeding is complex.
- Social and psychological variables such as maternal confidence, self‐efficacy and social support play a critical role.

**What are the key findings?**

- Maternal trait personality is associated with breastfeeding duration. Mothers who are introverted or have high levels of trait anxiety are less likely to initiate and continue breastfeeding.
- Maternal introversion and anxiety may affect women's ability to breastfeed successfully. These personality traits are associated with social and psychological variables known to predict low breastfeeding duration.

**How should the findings be used to influence policy/practice/research/education?**

- Considering maternal personality may be a useful tool to identify women who may need directed support.
- Although trait personality is considered fixed, exploring the impact of a mothers personality on her coping strategies may be useful.
- Interventions could encourage, for example, more introverted women to think about how they can increase their social networks in relation to breastfeeding.

## Introduction

Breastfeeding is established as beneficial to both infant and maternal health. Risks of gastroenteritis, respiratory infections, allergies and obesity are all increased among formula‐fed infants, while mothers who formula feed have greater levels of reproductive cancers (Kramer & Kakuma 2012, Ip *et al*. 2009). However, despite World Health Organisation's recommendations that infants are exclusively breastfed for the first six months postpartum with continued breastfeeding for up to two years and beyond (WHO 2002), breastfeeding rates in the UK are low. Although 81% of mothers now initiate breastfeeding at birth (McAndrew *et al*. 2012), by six weeks, rates have dropped to 48% with only 25% breastfeeding at all at six months (Bolling *et al*. 2007). Understanding influences on breastfeeding duration is critical to designing appropriate and effective interventions to support mothers to reach their breastfeeding goals.

### Background

The decision to breastfeed is complex with attitudes and concerns surrounding inconvenience, difficulty and embarrassment all influencing initiation (Wright *et al*. 2006, Brown *et al*. 2011a). Among mothers who wish to breastfeed, both physical and psychological factors affect breastfeeding duration (Thulier & Mercer 2009). Although true biological inability to breastfeed is rare (Huggins 2000), physical difficulties including latching the infant on, pain from nipple trauma, maternal exhaustion and perceived poor milk supply are often cited (David *et al*. 2007, Brown & Jordan 2012). Wider social factors also play a role including poor support from family and peers (McFadden & Toole 2006), negative partner attitudes (Li *et al*. 2008) and poor advice or conflict with health professionals (Brown *et al*. 2011b).

Maternal characteristics have also been implicated in breastfeeding duration. Maternal knowledge and understanding correlates well with breastfeeding duration (Spear 2006) with mothers who are proactive in seeking support and education feeding for the longest (Mitra *et al*. 2004, Heinig *et al*. 2006, Nelson 2007). Confidence (belief in your ability to achieve) is also critical, particularly in overcoming difficulties or responding to the criticism of others (Brown *et al*. 2011a). Self‐efficacy (belief in your ability to achieve a specific task at hand) also plays a central role (Kingston *et al*. 2007, Otsuka *et al*. 2008) as does self‐belief and determination to succeed (Avery *et al*. 2009). Interventions to increase breastfeeding self‐efficacy have led to increased levels of maternal self‐efficacy and trends towards longer breastfeeding duration and exclusivity (Nichols *et al*. 2007, McQueen *et al*. 2011). Overall, maternal confidence and self‐efficacy to breastfeed may be effective as it can encourage women to believe that they are able to breastfeed, to seek further professional support and to adopt a proactive stance to overcoming any issues faced (Blyth *et al*. 2002), all of which are associated with a longer breastfeeding duration (Thulier & Mercer 2009). Conversely, high levels of anxiety (Brown *et al*. 2011c), embarrassment (Andrew & Harvey 2011) and shyness (Flood & Dodgson 2010) appear to be associated with formula use.

Despite the known association between maternal characteristics and breastfeeding duration, there has been no empirical examination of the role of maternal personality on infant feeding decisions. Typically measured using a 5‐factor model of neuroticism, extraversion, agreeableness, conscientiousness and openness to experience (Costa & McCrae 1992), personality is considered to be biologically based, genetic and stable once adulthood is reached (Eysenck 1963). Personality inventories are widely used to explore and predict a variety of behaviours (Friedman *et al*. 2010). Indeed, personality has been related to several health issues including depression and anxiety (Cox *et al*. 2004) and behaviours such as smoking and weight gain (Hampson *et al*. 2006) and eating patterns (Hampson *et al*. 2007). Personality has also been shown to be related to several personal characteristics such as self‐efficacy, confidence and locus of control, which can in turn effect health outcomes (Vollrath 2001, Schaefer *et al*. 2004, Connor‐Smith & Flaschbart 2007).

The wide literature examining influences on breastfeeding shows that decisions about infant feeding are complex, including biological, psychological and social variables (Thulier & Mercer 2009). Given the association of personality with other health behaviours, maternal personality has the potential to play a role in influencing maternal infant feeding decisions, either directly or indirectly through personal characteristics. As this potential association between self‐efficacy and personality around breastfeeding attitudes, experiences and duration has not been explored in the literature, the aim of this study is to address this gap in our knowledge.

## The study

### Aim

The aim of this study was thus to explore associations among breastfeeding duration, maternal personality and maternal attitudes and experiences of breastfeeding. Understanding how maternal personality may play a role in affecting breastfeeding duration and experiences would further enable health professionals to identify and provide targeted support to new mothers.

### Design

This is an exploratory cross‐sectional survey.

### Participants

Data were collected between March–June 2009. Six hundred and two mothers living in the UK with an infant aged 6–12 months completed a self‐report questionnaire detailing maternal personality, breastfeeding duration and reasons for breastfeeding cessation if applicable. This age range was used to allow mothers to breastfeed for a significant duration as per World Health Organisation advice (e.g. potentially at least six months), but for recollection to be based on recent events (e.g. no longer than 12 months ago). Exclusion criteria included a low birthweight (<2500 g) and premature birth (<37 weeks).

Participants were recruited via local mother and baby groups in the Swansea area. These groups were located in areas with varying degrees of social deprivation as measured by the Welsh Indices of Multiple Deprivation (WIMD 2008). Posters were placed in venues advertising the study with details of how to contact the researcher for more details. In addition, questionnaires were distributed to mothers who attended groups via the group leader and returned to the centre or via post. Study adverts were also placed on online message boards on parenting forums based in the UK. Participants completed the questionnaire via an online link to the questionnaire.

Two hundred and eighteen (36·2%) completed the questionnaire using a paper copy with 386 (63·8%) using the online link. No significant difference was seen in mean age, years in education or breastfeeding duration between mothers who completed a paper or online version of the questionnaire.

### Data collection

Participants completed the Ten Item Personality Measure (Gosling *et al*. 2003) to measure the ‘Big‐five’ personality traits of Openness to Experience (Preference for novelty and variety and intellectual curiosity), Extraversion (Sociability, assertiveness and talkativeness), Agreeableness (Helpfulness, cooperation and sympathetic tendencies), Conscientiousness (Discipline, organization and achievement orientation) and Emotional Stability (anxiety and impulse control) (Goldberg 1993). Although this questionnaire is a short format personality measure, it shows strong convergence with more detailed versions, has high test–retest reliability and is considered a more valid measure than other short measures of personality (Furnham 2008).

Breastfeeding data were also collected. Participants indicated whether they initiated breastfeeding at birth and duration of any breastfeeding in days/weeks up to the current time point. Participants indicated whether they were still currently breastfeeding or not. Mothers were considered to be breastfeeding if they were doing so partially or exclusively.

Participants also completed a series of items examining attitudes towards breastfeeding (19 items) exploring issues such as health benefits, convenience and difficulty (items validated and based on previous work [Authors previous work (Table 1). Responses were based on 5‐point likert scales (strongly agree, agree, neither agree nor disagree, disagree, strongly disagree).

**Table 1:** Factor analysis of attitudes towards breastfeeding

	Difficult	Inconvenient	Formula content	Health
Breastfeeding is painful	**0·532**	0·265	0·103	−0·126
Lots of mums can't breastfeed	**0·721**	0·323	0·127	−0·222
Breastfeeding is difficult	**0·762**	0·185	0·131	−0·218
Lots of mums don't have enough milk	**0·734**	0·178	0·231	−0·127
Breastfeeding is exhausting	**0·621**	0·196	0·117	−0·312
You have to do all the feeds if you breastfeed	0·324	0·712	0·167	0·154
You can't go back to work if you breastfeed	0·117	**0·684**	0·212	−0·136
You can't have a social life if you breastfeed	0·104	**0·721**	0·182	−0·126
Only you can soothe the baby if you breastfeed	0·222	**0·612**	0·210	0·178
You are stuck in the house if you breastfeed	0·191	**0·545**	0·331	−0·218
Formula‐fed babies are more content	0·397	0·267	**0·555**	−0·110
Formula‐fed babies sleep better	0·300	0·231	**0·621**	−0·154
You have more of a routine if you formula feed	0·275	0·175	**0·634**	−0·113
Breastfed babies feed more often	0·254	0·152	**0·534**	0·114
Breastfeeding is best for babies health	0·312	−0·187	0·345	**0·789**
Breastfeeding is best for mothers health	−0·339	−0·275	0·421	**0·667**
Health professionals should encourage breastfeeding	−0·151	−0·138	0·317	**0·663**
% of variance	**35·12**	**12·37**	**5·15**	**3·49**
Cronbach's alpha	**0·792**	**0·781**	**0·702**	**0·846**

Table 1 shows regression scores for each item and how they load onto each factor produced. Items in bold signify items, which group strongly on each factor.

Previously used in Brown *et al*. 2011b,c, Brown & Jordan 2012.

If participants ceased breastfeeding before six months postpartum, they also completed a series of questions examining why (40 items) such as pain, embarrassment and exhaustion. This questionnaire had been previously used and validated in previous work (Authors previous work) (Table 2). Responses were based on 5‐point likert scales (strongly agree, agree, neither agree nor disagree, disagree, strongly disagree).

**Table 2:** Items and factor structure of questionnaire examining reasons for stopping breastfeeding

	Body image	Public Feeding	Difficulty	Pain	Lifestyle	Pressure	Support	Medical
Breastfeeding was ruining my breasts	**0·64**	0·13	0·05	0·12	0·23	0·05	0·01	0·25
I wasn't losing weight	**0·62**	0·31	0·32	0·25	0·15	−0·06	0·20	0·29
My breasts kept leaking	**0·62**	0·32	0·19	0·14	0·42	0·20	0·17	−0·02
I wanted my body back for me	**0·58**	0·18	0·03	0·15	−0·06	0·06	−0·01	−0·06
I didn't like feeding in public	0·10	**0·78**	0·22	0·14	0·20	−0·04	−0·01	−0·08
I didn't like feeding in front of others	0·01	**0·71**	0·09	0·25	0·06	−0·07	0·06	−0·32
I was stuck in the house breast feeding	0·30	**0·76**	−0·14	0·05	0·14	0·06	0·09	0·04
I didn't know anyone else who breast fed	0·04	**0·54**	0·47	0·05	0·06	0·25	0·19	0·18
The baby wouldn't latch on properly	0·24	0·17	**0·69**	0·10	0·04	0·17	0·13	0·12
The baby was feeding all the time	0·27	0·05	**0·80**	0·26	0·20	0·25	0·33	0·10
My baby wasn't gaining enough weight	−0·03	0·03	**0·67**	0·12	0·14	0·24	0·19	−0·09
I didn't have enough milk	−0·08	0·07	**0·64**	0·06	0·04	0·09	0·01	0·06
I couldn't breastfeed	0·08	0·08	**0·54**	0·28	0·06	0·20	0·22	0·01
I had a very hungry baby	0·27	0·12	**0·51**	0·07	0·07	0·20	0·12	0·04
Baby didn't want to breastfeed anymore	−0·07	0·09	**0·62**	−0·03	0·06	0·09	0·06	−0·03
It was too painful	0·05	0·16	0·02	**0·74**	0·25	0·10	0·07	−0·10
My nipples were cracked	0·11	−0·18	0·07	**0·69**	0·11	0·69	0·10	−0·01
I got mastitis, thrush or another similar problem	0·09	0·09	0·04	**0·72**	0·25	0·80	0·02	0·06
It was too difficult	−0·12	0·20	0·07	**0·84**	0·48	0·22	0·04	0·05
I never knew when the baby was going to feed	−0·01	0·08	−0·13	0·31	**0·78**	0·23	0·15	0·10
I didn't like being responsible for all the feeds	0·05	0·20	0·13	0·08	**0·64**	0·23	0·40	0·05
I couldn't keep track of milk intake	0·15	0·10	0·32	0·29	**0·65**	0·40	0·22	0·01
I couldn't leave the baby	0·04	−0·10	0·13	0·18	**0·59**	0·16	0·07	0·01
I couldn't go out and socialise	0·15	0·26	−0·05	0·02	**0·88**	−0·08	0·33	−0·06
I couldn't drink alcohol	0·20	0·08	0·05	−0·04	**0·78**	0·10	0·08	0·03
I wanted a more predictable routine	0·19	0·18	0·24	0·29	**0·68**	0·28	0·29	−0·12
I had breast fed for long enough	0·04	0·07	0·20	0·08	**0·72**	0·01	0·16	0·40
My partner wanted me to stop	0·02	0·20	0·08	0·02	0·20	**0·81**	0·39	0·13
My mother wanted me to stop	0·08	0·09	0·01	0·41	0·16	**0·76**	0·42	0·23
Friends wanted me to stop	0·02	0·26	0·19	0·06	−0·08	**0·66**	0·29	0·10
Other people made negative comments	−0·04	15	−0·05	0·12	0·10	**0·78**	0·45	0·11
Other people felt excluded	0·15	0·24	0·16	−0·05	0·04	**0·67**	0·25	0·13
I couldn't get any help with problems	0·10	0·32	0·22	−0·05	0·28	0·02	**0·82**	0·01
I didn't have enough support	0·47	0·18	0·19	0·04	0·28	0·19	**0·56**	0·20
I couldn't get any professional advice	0·08	0·32	0·54	0·13	0·11	−0·18	**0·63**	0·37
I was exhausted	0·40	0·13	0·72	0·21	0·03	0·15	**0·54**	0·28
I wasn't well	0·28	0·12	0·66	0·0	0·02	−0·02	0·22	**0·58**
The baby wasn't well	0·33	0·08	0·15	0·15	0·02	0·20	0·05	**0·78**
I was taking medication	0·05	0·29	0·18	0·41	0·19	−0·13	0·32	**0·62**
A health professional advised me to stop	0·22	0·28	−0·05	0·22	0·25	0·15	−0·11	**0·88**
Percentage of variance explained	**20·86**	**6·99**	**5·12**	**4·74**	**3·74**	**3·09**	**2·76**	**2·63**
Cronbach's alpha	**0·81**	**0·78**	**0·72**	**0·67**	**0·65**	**0·75**	**0·65**	**0·72**

Table 2 shows regression scores for each item and how they load onto each factor produced. Items in bold signify items, which group strongly on each factor.

Previously used in Brown *et al*. 2011b,c, Brown & Jordan 2012.

The decision was made to use these inventories as no suitable similar validated questionnaires could be found in the literature to measure the specific themes. Both questionnaires had been used in previous research (Authors previous work). Development of the original questionnaires was based on previous work using qualitative interviews to explore maternal experience of breastfeeding (Authors previous work) and recurring themes in the current literature as to why women cease breastfeeding, e.g. Li *et al*. (2008), Thulier and Mercer (2009). Questionnaires were originally piloted prior to the main study to test for usability (*n* = 20). No changes were made. Demographic information was also collected including maternal age, education, marital status and occupation.

### Ethical considerations

Full Research Ethics Committee approval was granted from a University Psychology research ethics committee. All applicable institutional and governmental regulations concerning the ethical use of human volunteers were followed. Participant study information and debrief were provided on written and online versions of the questionnaire with details of how to contact the researcher if more information was needed.

### Validity

Personality was measured using a validated tool (Furnham 2008, Gosling *et al*. 2003). Items in the breastfeeding questionnaire were based on recurring themes in the current literature (Li *et al*. 2008, Thulier & Mercer 2009) and preliminary qualitative interviews exploring influences on mothers' decisions to breast or formula feed (Authors previous work) and have been used in previous work (Authors previous work). Factor analysis was used to compute factors and Cronbach's alpha was used to examine internal consistency of the factors produced.

### Data analysis

The Ten Item Personality Measure was scored as per instructions to give the 5 scales of Openness to Experience, Extraversion, Agreeableness, Conscientiousness and Emotional Stability. Data were found to be normally distributed. Cronbach's alpha was computed for the 5 scales and was found to be high, ranging from 0·73–0·89.

For analyses using breastfeeding duration, a cut‐off was placed at six months (180 days) based on inclusion of participants in the sample with an infant age six months old. For these analyses, even if participants were still breastfeeding and their infant was aged over six months, only their breastfeeding duration up to six months old was considered.

Distribution of breastfeeding duration was abnormal (Kolmogorov–Smirnov = 0·238, *P* < 0·001) with a high proportion of mothers ceasing breastfeeding in the first few days and weeks or breastfeeding for a longer duration. Therefore, breastfeeding duration data were transformed and the natural logarithms computed were used to correct for the skewed distribution.

To examine the data related to breastfeeding attitudes and cessation, exploratory factor analysis was conducted on the two sets of items to statistically group items into key themes. Although the questions had been used in previous research, this analysis was conducted to ensure greater reliability of grouping items. Factor analysis is a statistical technique that combines large numbers of variables together into a smaller number of factors based on similarities in the variables. Using SPSS, a principal components factor analysis using varimax rotation was performed, retaining factors with eigenvalues over 1. A threshold of 0·5 was used to determine which variables should be retained. Further analyses performed on split samples of the data for confirmation found similar structures. The factor scores computed were saved as regression scores and used for the data analysis (Tabachnick & Fidell 2006). Cronbach's alpha was computed for each factor to examine internal consistency of the factors produced.

MANCOVA were then used to examine differences in maternal personality for those who breast or formula fed at birth. Spearman's correlations were used to examine association among maternal personality, breastfeeding duration, attitudes towards breastfeeding and reasons for cessation. Once analyses had been performed, breastfeeding duration data were back transformed to present logical mean duration scores. This approach was used rather than considering a non‐parametric measure as parametric tests are more powerful and elements such as effect size can be calculated (Tabachnick & Fidell 2006).

## Findings

Six hundred and two mothers completed the questionnaire (meaning that the analysis was sufficiently powered at 80% β and 0·05 α). Mean age of the respondents at childbirth was 29·16 years (range from 16–45), mean number of years in education was 14·61 and 72·3% of mothers were primiparous. Demographic spread of the data can be found in Table 3.

**Table 3:** Sample distribution by Demographic Factors

Indicator	Group	*N*	%
Age	≤19	14	2·2
	20–24	124	19·6
	25–29	173	27·2
	30–34	180	28·4
	35≥	142	22·6
Education	No formal	25	3·9
	School	175	27·5
	College	162	25·5
	Higher	271	42·8
Marital Status	Married	336	53·1
	Cohabiting	197	31·3
	Single	99	15·6
Home	Owned	349	55·1
	Rented	244	38·4
	Council	85	13·5
	Other	4	0·8
Maternal occupation	Professional & managerial	210	33
	Skilled	126	19·8
	Unskilled	80	12·6
	Other	29	4·6
	Stay at home mother	190	42·5

### Breastfeeding and maternal personality

One hundred and one mothers formula fed from birth (16·7%), whereas 501 (83·3%) breastfed. Two hundred and seventeen mothers breastfed for at least six months postpartum (36·0%), whereas 284 mothers initiated breastfeeding, but stopped before six months postpartum (range two days–12 weeks). Of this sub‐group, 58·5% of mothers stopped breastfeeding within the first week postpartum with 73·2% having stopped by two weeks. Thus, data were transformed to correct skewed distributions.

A Multivariate ancova was used to compare differences in maternal personality for mothers who breast or formula fed at birth controlling for maternal age, education and parity (Table 4). Significant differences were found for maternal extraversion [*F* (1, 600) = 11·54, *P* = 0·001], emotional stability [*F* (1, 600) = 5·616, *P* = 0·018] and conscientiousness [*F* (1, 600) = 3·855, *P* = 0·048]. Mothers who breastfed at birth reported significantly higher levels of extraversion, emotional stability and conscientiousness. No significant difference was found for openness to experience and agreeableness.

**Table 4:** Breastfeeding and personality traits: Showing mean personality scores (and standard deviation) at specific time points postpartum

Time postpartum	Any Breastfeeding	*N*	Extraversion	Emotional Stability	Openness	Conscientiousness	Agreeableness
Birth	Yes	501	8·45 (2·44)**	7·56 (2·63)**	6·88 (2·58)	8·51 (2·32)*	9·12 (2·16)
	No	101	7·33 (2·49)**	6·79 (2·40)**	6·33 (2·56)	8·78 (2·34)*	8·78 (2·16)
Two weeks	Yes	320	8·41 (2·42)**	7·50 (2·63)**	6·94 (2·57)	8·42 (2·47)	8·75 (2·47)
	No	228	7·39 (2·50)**	7·01 (2·56)**	6·62 (2·59)	8·70 (2·14)	8·93 (2·03)
Six weeks	Yes	229	8·55 (2·29)*	7·79 (2·54)**	7·00 (2·61)	8·74 (2·45)	8·91 (2·23)
	No	373	7·77 (2·54)*	7·03 (2·62)**	6·66 (2·57)	8·44 (2·24)	8·79 (2·12)
Twelve weeks	Yes	169	8·03 (2·08)	7·69 (2·77)*	6·66 (2·51)	8·68 (2·47)	9·01 (2·15)
	No	433	8·25 (2·60)	6·88 (2·08)*	6·84 (2·61)	8·56 (2·26)	8·86 (2·16)
Twenty six weeks	Yes	155	8·25 (1·99)	7·51 (2·78)*	6·69 (2·53)	8·77 (2·46)	9·01 (2·18)
	No	447	8·00 (2·61)	6·87 (1·93)*	6·82 (2·60)	8·54 (2·26)	8·87 (2·15)

Shaded areas represent significant difference in personality trait between feeding groups as per MANCOVA test. *P < 0·001; **P < 0·05.

Differences in maternal personality traits were also examined for breastfeeding duration considering any breastfeeding at two, four, six, 12 and 26 weeks (Table 4). Mothers who were still breastfeeding at each of these time points were rated significantly higher in emotional stability at all time points and significantly higher in extraversion at two, four and six weeks. No significant difference in openness to experience, conscientiousness or agreeableness was seen at any postnatal time point.

### Maternal Personality and Attitudes towards breastfeeding

Principal components factor analysis was performed on all items examining attitudes towards breastfeeding producing five factors and explaining 56·13% of the variance (Table 1). Factors were labelled ‘difficult’ (painful, exhausting), inconvenient (interfering with maternal lifestyle, placing greater responsibility on the mother than formula feeding), ‘formula fed infants are more content’ (believing formula fed infants to be easier to settle and sleep for longer) and ‘breastfeeding as healthier’ (benefits for infant and maternal health). Regression scores for each factor were computed and used for comparison. Cronbach's alpha was also computed for each factor, ranging from 0·65–0.81, and is also shown in Table 1. Although this questionnaire had been used in existing research (Authors previous work) and showed medium‐to‐high reliability scores, the decision was made to re‐compute cronbach's alpha here to show high retest reliability of the measure.

Partial spearman's rho correlations examined association between attitudes and maternal personality controlling for breastfeeding duration (Table 5). Extraversion was significantly inversely associated with believing breastfeeding to be difficult (Spearman's rho = −0·109, *P* = 0·041). Emotional stability was significantly inversely associated with believing breastfeeding to be difficult (Spearman's rho = −0·581, *P* < 0·001), but positively significantly associated with believing breastfeeding to be healthier (Spearman's rho = 0·451, *P* < 0·001). Finally, Conscientiousness was significantly positively associated with believing breastfeeding to be healthier (Spearman's rho = 0·672, *P* < 0·001) and inversely associated with believing breastfeeding to be inconvenient (Spearman's rho = 0·115, *P* = 0·008).

**Table 5:** Correlations among personality factors, attitudes and experiences

	Extraversion	Emotional stability	Conscientiousness	Openness to experience	Agreeableness
Attitudes					
Difficult	−0·688 (0·000)**	−0·581 (0·000)**	0·036 (0·195)	0·064 (0·098)	0·060 (0·076)
Inconvenient	−0·006 (0·440)	0·020 (0·316)	−0·115 (0·014)*	−0·046 (0·139)	−0·043 (0·152)
Content	0·044 (0·145)	0·011 (0·397)	−0·052 (0·106)	0·008 (0·421)	−0·016 (0·348)
Health	0·032 (0·225)	0·451 (0·000)**	0·672 (0·000)**	−0·004 (0·467)	−0·012 (0·389)
Reasons for stopping					
Difficult	0·022 (0·364)	−0·155 (0·006)**	−0·012 (0·424)	−0·019 (0·453)	0·025 (0·344)
Feeding in public	−0·112 (0·037)*	0·012 (0·143)	−0·034 (0·293)	−0·019 (0·453)	0·060 (0·168)
Body image	0·070 (0·131)	0·044 (0·243)	−0·109 (0·041)*	−0·061 (0·165)	−0·025 (0·344)
Pain	0·071 (0·129)	−0·009 (0·445)	−0·085 (0·045)*	0·038 (0·275)	−0·011 (0·432)
Lifestyle	0·013 (0·421)	0·009 (0·445)	−0·065 (0·149)	0·088 (0·079)	0·017 (0·396)
Pressure from others	−0·103 (0·005)*	−0·049 (0·442)	−0·009 (0·442)	−0·055 (0·192)	0·035 (0·288)
Lack of support	−0·089 (0·077)	−0·120 (0·028)*	−0·094 (0·067)	−0·017 (0·390)	0·040 (0·260)
Medical reasons	0·022 (0·235)	0·083 (0·092)	−0·033 (0·297)	0·103 (0·051)	−0·042 (0·253)

Shaded boxes show significant associations *P < 0·05; **P < 0·01.

### Maternal Personality and Reasons for breastfeeding cessation

Using the same method as described above, principle components analysis was performed on all items examining reasons for breastfeeding cessation. The model explained 49·93% of the variance with strong Cronbach's alpha (Table 2). Factors were labelled body image concerns (worries about appearance and leaking milk), Public Feeding (not wanting to feed in front of others or in public), difficulty (problems with latch and positioning), pain (from cracked nipples or mastitis), impact on lifestyle (lack of routine and difficulties socializing), pressure from others to stop (from friends, family and partner), lack of support (difficulties getting advice or support with problems) and medical reasons (taking medication or advised to stop by a professional). Regression scores were computed and used for analysis.

As extraversion, emotional stability and conscientiousness were the three traits significantly associated with breastfeeding duration, examination of the relationship between these variables and reasons for cessation were examined. This section reports findings from mothers who initiated breastfeeding, but stopped before 6 months postpartum (Table 5).

Extraversion was significantly inversely associated with ceasing breastfeeding due to issues with public feeding (Spearman's rho = −0·112, *P* = 0·037) or feeling pressured by others to stop (Spearman's rho = −0·103, *P* = 0·005). Thus, mothers who were more introverted were more likely to have found breastfeeding embarrassing or felt pressured to stop breastfeeding. Emotional stability was significantly inversely associated with reporting stopping breastfeeding due to a lack of support with problems (Spearman's rho = −0·120, *P* = 0·028) or difficulty (Spearman's rho = −0·155, *P* = 0·006). Mothers who were more anxious reported greater difficulty and lack of support. Conscientiousness was significantly inversely associated with reporting stopping breastfeeding due to body image concerns (Spearman's rho = −0·109, *P* = 0·041) and pain (Spearman's rho = −0·085, *P* = 0·018). Lower conscientiousness was therefore associated with greater issues with pain and body image.

## Discussion

This paper examined associations between maternal personality and breastfeeding duration, considering the role of variations in attitudes and experiences in explaining any relationship. Mothers who reported higher levels of emotional stability, extraversion and conscientiousness were significantly more likely to initiate and continue breastfeeding, potentially due to being more confident in their approach to breastfeeding. Although maternal characteristics such as anxiety, confidence and self‐efficacy have been explored in relation to breastfeeding duration, as far as we are aware, this is the first paper to examine the issue of understanding breastfeeding from a trait personality model. These findings are of interest as not only do they add to the literature exploring the public health issue of increasing breastfeeding duration, but the simplicity of the measure used gives opportunity for the importance of maternal personality to be considered in an applied setting.

The main limitations of this study surround the self‐selecting sample, which may have led to only the most motivated women participating, e.g. a particular interest in breastfeeding or particular difficulties. Indeed, a larger proportion of the sample did breastfeed for at least 6 months compared with other UK surveys such as the Infant Feeding Survey (e.g. 36% vs. 25%). However, initiation and early continuation rates were similar (Bolling *et al*. 2007). Related to this, the sample was weighted towards an older, more educated demographic, although the sample was varied in terms of demographic background due to targeted recruitment in more deprived areas. The sample was also predominantly White British in ethnic origin (95·6%). Generalizability must, however, be undertaken with caution.

The retrospective design of the questionnaire is also a limitation. This approach was used due to the novel exploration of the area, but criticism could be made that using maternal self‐reported recall of breastfeeding duration is inaccurate. However, a retrospective approach has been used successfully in several other studies exploring health outcomes (Felitti *et al*. 1998, Brunstrom *et al*. 2005, Brown & Lee 2011) and the time period for recall was short. Examination of the accuracy of retrospective reports also suggests that they are reliable (Brewin *et al*. 1993). With regard to retrospective measures of personality, personality is generally considered to be a stable trait (Furnham 2008) with suggestion that maternal personality traits are stable throughout the perinatal period (Grant *et al*. 2008). However, further research might benefit from a prospective or even longitudinal approach.

Criticism could also be made of the short personality measure used. However, the Ten Item Personality Measure shows strong reliability and validity and is comparable to outcomes of more detailed questionnaires (Gosling *et al*. 2003, Furnham 2008). Further examination may wish to use a more detailed measure, but this would increase the level of input needed from participants. It could also be argued that although trait personality is considered to be stable during adulthood (Ferguson 2010), the perinatal period can have an impact on maternal identity, purpose and networks (Nelson 2003). Risk of mood disorders is also increased during this period (Cohen & Nonacs 2005). However, women were not in the immediate perinatal period and there is growing evidence that maternal personality traits are stable through pregnancy and the postnatal period (Grant *et al*. 2008). Indeed, trait measures have been used in several studies during the perinatal period (e.g. McMahon *et al*. 2001, Hart & McMahon 2006).

Overall, significant associations were found between breastfeeding initiation and duration and maternal trait emotional stability, extraversion and conscientiousness. These traits in turn were also significantly associated with patterns in attitudes towards breastfeeding and reasons for cessation. It is arguable that maternal beliefs and behaviours potentially associated with their personality are enabling or thwarting their likelihood of breastfeeding.

Specifically, mothers with a more introverted personality believed breastfeeding to be more difficult than those who had stronger extraverted tendencies. Breastfeeding, although natural, is a skill, which can take time to master with many mothers ceasing breastfeeding due to difficulties getting the infant to latch on, pain from cracked nipples or concerns about milk supply (Scott *et al*. 2006, Gatrell 2007, David *et al*. 2007). Personality type is associated with variation in coping style when facing difficulties. Extraverts are more likely to seek support from others (Williams & Galliher 2006), adopt a problem focussed coping response (Connor‐Smith & Flaschbart 2007) and feel that they have the ability to cope with a situation (Vollrath 2001). Potentially extraverted mothers adopt a more proactive approach, behaving in ways that enhance breastfeeding success such as attending antenatal classes (Donath & Amir 2003) or seeking specialist support and information (Nelson 2007).

Higher levels of introversion were also associated with feeling pressurized by others to stop breastfeeding. Many mothers in the UK feel that they live in a formula‐feeding culture, where breastfeeding is not the normative choice (McFadden & Toole 2006). A lack of understanding from others or pressure to stop breastfeeding especially when problems occur is common (Thulier & Mercer 2009). Examining the role of personality here, extraverts are more likely to display assertion (Rothbart & Hwang 2005), self‐efficacy (Schaefer *et al*. 2004) and confidence (Keller *et al*. 2011), which may enable women to challenge the views of others who suggest that they should stop breastfeeding. Evidence is growing that interventions designed to increase maternal self‐efficacy to breastfeed not only have a positive impact on maternal belief and confidence that she can breastfeed her baby, but are associated with trends towards increased breastfeeding duration and exclusivity (Nichols *et al*. 2007, McQueen *et al*. 2011). Potentially, working to improve the self‐efficacy of mothers who present with an introverted or anxious personality type may enable them to breastfeed for longer.

Finally, mothers who were more introverted were more likely to report that they stopped breastfeeding because they felt embarrassed. Feeling embarrassed about feeding in front of others is associated with a shorter breastfeeding duration (Khoury *et al*. 2005, Nelson & Sethi 2005). Again, this is likely to be linked to feelings of confidence and self‐efficacy that are lower among introverts.

Breastfeeding duration was also associated with emotional stability. Low confidence can lead to formula use (Forster *et al*. 2006) including specific anxieties regarding low milk production (Li *et al*. 2008), slow weight gain (Sachs *et al*. 2006) or concern that the infant is not receiving enough milk (Brown *et al*. 2011c). Anxiety can be categorized as either ‘state anxiety’ (transient and related to a particular time or situation or ‘trait anxiety’ (a stable personality difference in anxiety proneness) (Goldberg 1993). These findings suggest that stable trait anxiety may also be playing a role alongside more specific transient concerns about milk supply and weight gain.

The associated attitudes with emotional stability may help explain this link. Mothers who were more anxious reported greater difficulty and lack of support. It would be interesting to explore the direction between these factors more clearly. Is maternal trait anxiety associated with actual higher levels of difficulties or rather a perception of more difficulties? Mothers who are more anxious may perceive difficulties to be greater or to feel that they are not being supported to the level that they need. Neuroticism has been linked to increased pessimism (Williams 1992), greater perceived threat (Suls & Martin 2005), increased distress and fear (Rothbart & Hwang 2005) and lower self‐efficacy (Ebstrup *et al*. 2011), suggesting that mothers low in emotional stability may be at greater risk of the negative combination of becoming overwhelmed and not seeking support.

Finally, individuals who were more conscientious were more likely to initiate, but not continue to breastfeed. Individuals who are high in conscientiousness are more likely to follow health guidance, for example abstaining from smoking, being at a healthy BMI and eating more healthily in general (Hampson *et al*. 2006, 2007). Indeed, conscientiousness was significantly linked to the belief that breastfeeding was healthiest, thus potentially increasing motivation to breastfeed. What may be happening here, however, is that high levels of conscientiousness increase mothers desire to breastfeed, but may not enable them to continue. Believing breastfeeding to be best for the infant, but feeling unable to do so, is linked to high levels of guilt (Lee 2011). Support needs to be directed to enabling and educating women.

Overall, the findings show a novel and interesting link between breastfeeding duration and maternal trait personality adding to the literature examining the role of personality and health behaviours and outcomes (Cox *et al*. 2004, Hampson *et al*. 2006, 2007). Potentially, this knowledge could enable health professionals and those working in breastfeeding support to provide more targeted support. The Ten Item Personality Measure is a brief measure that could be used either individually in antenatal care or in group sessions as a discussion trigger for considering issues around breastfeeding and encouraging proactive support and information seeking.

### Limitations

'However, wider consideration needs to be given to the complex social and psychological influences on breastfeeding duration (Thulier & Mercer 2009). Personality is certainly not the only influence on infant feeding behaviour and considering it as such, in light of its biological construct, is a reductionist view. However, maternal personality measurement may play an important role in understanding a woman's wider experiences and social environment. Assessing maternal personality is a simple and brief tool that could alert health professionals to potential wider issues a woman might face.

For example, trait personality is typically considered to be a biologically based, genetic entity that remains stable once adulthood is reached (Eysenck 1963). If breastfeeding duration is linked to this static trait, this information alone is of little use to health professionals working to support new mothers with breastfeeding. However, the findings of this study show that personality was associated with certain attitudes and experiences with regard to breastfeeding. For example, mothers with lower emotional stability reported lower levels of emotional support and greater difficulty, whereas introverts appeared to be more affected by the negative attitudes of others. These factors are modifiable and indeed, interventions to increase maternal confidence, knowledge and support networks through peer support systems of self‐efficacy have been effective (e.g. Kramer *et al*. 2001, Stockdale *et al*. 2008). Thus, a simple measure of personality may be useful in raising awareness of mothers who may need further psychosocial support in initiating and maintaining breastfeeding.

## Conclusion

Limitations aside this study raise the issue for the first time that maternal personality may play a role in the complex array of factors influencing maternal infant feeding decisions. Understanding the relationship between maternal characteristics of extraversion, emotional stability and conscientiousness and the impact these may have on breastfeeding duration could enable health professionals to further target their support.

## Funding

Supported by an ESRC Postdoctoral Fellowship.

## Conflict of interest

No conflict of interest.

## Author contributions

All authors have agreed on the final version and meet at least one of the following criteria [recommended by the ICMJE (http://www.icmje.org/ethical_1author.html)]:

- substantial contributions to conception and design, acquisition of data, or analysis and interpretation of data;
- drafting the article or revising it critically for important intellectual content.
